# Behavioral and Cognitive Problems as Determinants of Malnutrition in Long-Term Care Facilities, a Cross-Sectional and Prospective Study

**DOI:** 10.1007/s12603-022-1827-3

**Published:** 2022-07-26

**Authors:** Jos W. Borkent, H.P.J. van Hout, E.J.M. Feskens, E. Naumann, M.A.E. de van der Schueren

**Affiliations:** 1 HAN University of Applied Sciences, Kapittelweg 33, 6525 EN, Nijmegen, The Netherlands; 2 Amsterdam University Medical Center, Vrije Universiteit, Depts. of General Practice and Medicine for Older Persons, Van der Boechorsstraat 7, 1081 BT, Amsterdam, The Netherlands; 3 Wageningen University, Stippeneng 4, 6708 WE, Wageningen, The Netherlands

**Keywords:** Undernutrition, cognition, nursing homes, residentialcare, longitudinal research

## Abstract

**Objectives:**

To investigate the cross-sectional and prospective associations between behavior and cognitive problems and malnutrition in long-term care facilities (LTCF).

**Design:**

Cross-sectional and prospective routine care cohort study.

**Setting:**

6874 Residents in Dutch LTCFs (period 2005–2020).

**Participants:**

Data were obtained from the InterRAI-LTCF instrument. Cross-sectional analyses on prevalence of malnutrition at admission included 3722 residents. Prospective analyses studied incident malnutrition during stay (total follow-up time 7104 years) and included data of 1826 residents with first measurement on admission ('newly-admitted') and n=3152 with first measurement on average ∼1 year after admission ('existing').

**Measurements:**

InterRAI scales for communication problems (CS), aggressive behavior (ABS), social engagement (RISE), depressive symptoms (DRS), cognitive performance (CPS) and the total number of behavior and cognitive problems were investigated as independent variables and malnutrition (ESPEN 2015 definition) as dependent variable in regression analyses. Results were stratified for gender and group ‘newly-admitted’ vs. ‘existing’.

**Results:**

On admission, 9.5% of residents was malnourished. In men, low social engagement was associated with prevalence of malnutrition. In women, all behavior and cognitive problems except depression were associated with malnutrition in the unadjusted analyses, but this attenuated in the full model taking all problems into account. The incidence of malnutrition during stay amounted to 8.9%. No significant associations of behavior and cognitive problems with malnutrition incidence were seen in ‘newly-admitted’ male residents while in ‘existing’ male residents all determinants were significantly associated. In ‘newly-admitted’ female residents CS, ABS and CPS, and in ‘existing’ female residents CS, RISE, ABS and CPS were significantly associated with incident malnutrition. All associations slightly attenuated after adjustment. Malnutrition incidence increased with increasing number of combined behavior and cognitive problems.

**Conclusion:**

Residents with behavior and cognitive problems are at an increased risk of being malnourished at admission, or becoming malnourished during stay in a LTCF, especially residents with multiple behavior and cognitive problems.

## Abbreviations

LTCF: Long-term care facility
MDS: minimum data set
BMI: body mass index
CPS: cognitive performance scale
CS: communication scale
RISE: revised index of social engagement
DRS: depressive rating scale
ABS: aggressive behavior scale

## Background

**T**he European population of 65 years and older is expected to increase from 19% nowadays, towards 28.5% in 2050 (1). Many European countries encourage ‘ageing-in-place’, whereby most older adults remain living in their own home and community with or without (informal) care (2). Ageing-in-place enables older adults to remain as autonomic, independent and (socially) active as possible (3, 4). Based on this policy, older adults only move to residential care facilities when their level of dependency is high and home-care is no longer sufficient (5).

Older adults with severe behavior and cognitive problems become affected in their (instrumental) activities of daily living (ADL) (6, 7), and this puts a high burden on formal and informal care at home (8). When people with advanced behavior and cognitive problems become fully care-dependent, they are more likely to become institutionalized (8, 9, 10).

While still living at home, the ability to perform grocery shopping and prepare meals is one of the first functions that is lost in older adults with cognitive decline, herewith increasing the risk of impaired food intake (6). Previous cross-sectional studies have shown a clear association between cognitive impairment and the presence of malnutrition, both in the community and during stay in a long term care facility (LTCF) (11, 12, 13, 14, 15). However, studies describing incident malnutrition during stay in a LTCF in residents suffering from behavior and cognitive problems are lacking.

We hypothesize that residents with behavior and cognitive problems are at increased risk to be already malnourished at admission to a LTCF, or to become malnourished during stay. Therefore, the aim is of this study is to describe the cross-sectional and long-term associations between behavior and cognitive problems at admission, malnutrition prevalence at admission and incident malnutrition during stay in a LTCF in a large population.

## Methods

### Data source

Data were obtained from InterRAI; a non-profit international multidisciplinary collaboration that aims to improve quality of life of older adults through systematic, accurate and standardized data collection of residents' physical and psychosocial functioning (16). A specific assessment form was developed for each healthcare setting (17). The RAI Long-Term Care Facilities (LTCF) assessment form is a minimum data set (MDS) which includes nineteen sections, including residents' nutritional, cognitive and psychosocial status. The assessment form is administered by trained nurses in interaction with the residents, their family members and other health professionals (17). Several studies have shown high validity and reliability of the assessment form (18, 19, 20).

Two groups were derived from residents admitted to a LTCF: the ‘newly admitted’ and the ‘existing’ residents. The newly admitted group had their first assessment taken place within one month after admission to a LTCF (‘admission assessment‘); thereafter residents were monitored typically with quarterly or semi-annual follow-up assessments.

When a LTCF started using InterRAI, no ‘admission assessment’ was performed for the residents who were already living in that facility (‘existing’ residents). A ‘delayed first assessment’ was then noted as first measurement.

Dutch InterRAI subjects (aged ≥ 65 years) living in LTCF between 2005 and 2020 were included in this study. In total, 4190 residents with an ‘admission assessment’ were available, median time to first assessment was 16 days [IQR: 7–30] after admission. These residents represent ‘newly-admitted’ residents.

In contrast, the ‘existing’ residents are defined by having had their first measure after their initial admission. In total 5592 residents with a ‘delayed first assessment’ were available and this assessment took place with a median time of 345 [IQR: 117–914] days in male and 546 [IQR: 165–1363] days in female residents after their initial admission. Thus, the defining difference between the groups is the time elapsed between their admission and their first assessment, which was shorter in the newly admitted group and longer in the ‘existing’ residents' group.

### Inclusion criteria cross-sectional analyses

For the cross-sectional analyses, only data of ‘admission assessments’ were used (n=4190). Residents were included if data regarding malnutrition status were available. Exclusion criterion was presence of end-stage disease, i.e. terminally ill residents with a life expectancy <6 months as indicated by the treating physician, to exclude residents with incurable malnutrition. After exclusion, 3722 residents were available for analyses.

### Inclusion criteria prospective analyses

For the prospective analyses, first assessment data of ‘newly-admitted’ residents as well as first assessment data of ‘existing’ residents and all subsequent follow-up measurements were used. Residents were included when they were not malnourished at their first available measurement and had one or more follow-up measurements where nutrition status was measured. Exclusion criterion was presence of end-stage disease at first or last measurement. After exclusion, 4978 residents were available for analyses.

**Figure 1:**
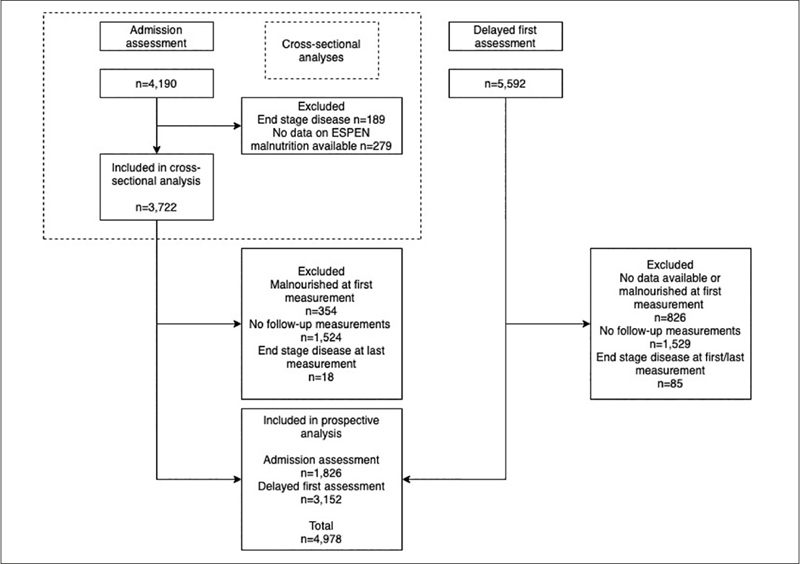
Flowchart of included participants in cross-sectional and prospective analyses

Figure 1 provides an overview of the in- and exclusion process.

### Measurements

We used the following InterRAI scales for behavior and cognitive problems as independent variables: Communication Scale (SC), Cognitive Performance Scale (CPS), Depression Rating Scale (DRS), Revised Index of Social Engagement (RISE), Aggressive Behavior Scale (ABS) and total number of behavioral-cognitive problems. Malnutrition based on the ESPEN 2015 criteria was used as dependent variable.

### Communication Scale (CS)

The CS is a standardized questionnaire that assesses the communication performance of subjects living in a LTCF. It consists of two communication items (understanding others, making oneself self-understood). The scale provides a score ranging from 0 (good communication performance) till 8 (poorest communication performance) (21). No validated cut-off values are available for CS but previous research showed that a cut-off value of ≥3 will identify the 10% most severe cases with communication problems (22). The CS was dichotomized into good communication performance (CS ≤2) or moderate to severe impairment (CS ≥3).

### Cognitive Performance Scale (CPS)

The CPS is a standardized and validated questionnaire containing five items (decision making, memory, disordered thinking, change in mental status, change in decision making) to assess cognitive performance of subjects living in a LTCF (23, 24). It provides a total score ranging from 0 (cognitive performance intact) till 6 (very severe cognitive impairment) (24). The CPS has been validated against the MMSE, whereby a CPS score of 2 equals a MMSE score of 19 (24). Therefore, the CPS scale was dichotomized into ≤2 and ≥3.

### Depression Rating Scale (DRS)

The DRS is a standardized and validated screening questionnaire to screen for depressive symptoms in subjects living in a LTCF (25, 26). It includes seven depressive mood and behavioral indicators (negative statements, anger, unrealistic fears, health complaints, anxious complaints, sad expressions, crying). All indicators have possible scores of 0 (indicator not present during the past 30 days), 1 (indicator present 1 till 5 times a week) or 2 (indicator present 6 or 7 days a week), resulting in a total maximum score of 14. A total score ≥ 3 indicates a resident is at risk for depression (26). Therefore, this item was dichotomized in low (≤2) or high depression risk (≥3).

### Revised Index of Social Engagement (RISE)

The RISE is a standardized and validated measure of social engagement of subjects living in a LTCF (27). It includes six dichotomous indicators of social engagement (initiating social interaction, accepting social interaction, activity participation, accepting invitations and facility involvement). The scale provides a score ranging from 0 (poor social engagement) till 6 (high social engagement) (27). Based on the original validation paper of RISE, a cut-off of ≤2 reflects a division between low- and high functioning people (28). Therefore, the RISE was dichotomized into low social engagement (RISE score ≤2) and high social engagement (RISE score ≥3). The high social engagement group (RISE score ≥3) was chosen as reference group in our analysis.

### Aggressive behavior scale (ABS)

The ABS is a validated four-item scale based on the following items: verbal abuse, physical abuse, socially inappropriate behavior and resisting care (29). These items are coded as: not present (0), present in last three days (1), happened once or twice in three days (2) or daily (3). Based on the four items, scores range from 0–12, and higher scores indicate aggressive behavior. The ABS was dichotomized into no aggressive behavior (ABS=0) or aggressive behavior present (ABS ≥1) (29).

### Total number of behavior and cognitive problems

Based on the dichotomized scores of the scales mentioned above, a sum score of CS, CPS, DRS, RISE, ABS was created, which indicated on how many of the behavioral-cognitive scales problems were identified (range 0–5).

### Malnutrition

The primary end point of this study was malnutrition based on the ESPEN 2015 criteria; Body Mass Index (BMI) < 18.5 kg/m^2^, or weight loss (5% during last month or 10% in six months), in combination with a reduced age-specific BMI (< 20 kg/m^2^ < 70 years or < 22 kg/m^2^ ≥ 70 years) (30).

### Covariables

Data on age, gender, living status before admission (together vs. alone) and number of underlying diseases were obtained from the RAI-LTCF Assessment Form.

### Ethical considerations

InterRAI assessments are performed for clinical purposes as part of routine care. Data is de-identified and thereafter transferred to the InterRAI database at the Amsterdam University Medical Centres — Location VUmc. Residents are informed (by their practice nurse, through newsletters, posters and website) in general terms that their data can be used for research purposes. Residents can object against use of their data and an opt-out procedure is available therefore. The Ethical committee of VUmc approved the use of data for research in this way.

### Statistical analysis

All statistical analyses were performed in SPSS version 25 (IBM Corp., Armonk, New York, USA). Descriptive statistics were stratified by nutritional status and gender. Normality was checked by QQ-plots and stem-and-leaf plots. Means with standard deviations were used to describe continuous variables and numbers and percentages for categorical data. Logistic regression analyses were used to study the associations between CPS, CS, DRS, RISE, ABS and total number of behavior and cognitive problems (independent variables), and malnutrition (dependent variable). Kaplan-Meier curves and Cox Proportional Hazard regression analyses were performed with malnutrition as event and CPS, CS, DRS, RISE, ABS, and total number of behavior and cognitive problems as independent variables. Time to event was defined as days between first available assessment and the first follow-up assessment where malnutrition occurred. If someone was categorized as malnourished, all further follow-up measurements were removed. Residents who stayed well-nourished during their total follow-up period were censored and event time ended at their latest available measurement. Kaplan-Meier curves provided a graphical evaluation of the proportional hazard assumption. As these assumptions were met, Cox Proportional Hazard regression analyses were performed.

Stratification for gender was based on the significance of interaction terms (behavior-cognitive problem * gender). In the cross-sectional analyses this interaction term was significant (p<0.10) (31) for RISE, CPS and total number of behavior-cognitive problems. In the prospective analyses, interaction terms for DRS and total number of behavior-cognitive problems were statistically significant.

Thereafter, we tested whether there was effect modification by type of first assessment in the prospective analyses (behavior-cognitive problem * type of first assessment). Significant interaction terms (p<0.10) (31) were seen for male gender (CS, DRS, ABS, CPS and total number of behavior-cognitive problems). Based on this number of significant interaction terms and the large differences in effect sizes between men/women and newly-admitted/existing residents, we decided to present four different strata (for illustration, see appendix 1 for additional Kaplan-Meier curves on CS).

As previous studies showed that malnutrition is related to very old age (≤ 90 years vs. ≥ 91 years) (32), number of comorbidities (≤1 vs. ≥2) (33, 34) and living status before admission (alone vs. together) (35), all regression analyses were adjusted for these variables (model 1). As most behavioral-cognitive problems are associated with each other, a full model was created which included in addition all five determinants (CS, DRS, RISE, ABS and CPS) (full model).

## Results

### Cross-sectional analysis

Table 1 shows the characteristics of the 3722 included residents at LTCF admission. Most residents were women (67.6%), lived alone before admission (64.3%), were aged <90 years (81.9%) and their BMI was within the normal range (mean 24.7 kg/m2, SD 4.6). Prevalence rates of behavioral-cognitive problems ranged from 22.4% for aggressive behavior (ABS) to 27.8% for depression risk (DRS).

**Table 1:** Characteristics of included participants at admission to LTCF, stratified by gender and nutritional status

	**Male**		**Female**		**Total**
	**N=1207 (32.4%)**		**N=2515 (67.6%)**		**N=3722**
	**Malnourished**	**Well-nourished**	**Malnourished**	**Well-nourished**	
	**N=88 (7.3%)**	**N=1119 (92.7%)**	**N=266 (10.6%)**	**N=2249 (89.4%)**	
Age (years)	82.8 (SD:6.8)	81.8 (SD:7.2)	84.3 (SD:7.0)	83.7 (SD:6.9)	83.1 (SD:7.1)
≤ 89 years	75 (85.2%)	963 (86.1%)	197 (74.1%)	1815 (80.7%)	3050 (81.9%)
≥ 90 years	13 (14.8%)	156 (13.9%)	69 (25.9%)	434 (19.3%)	672 (18.1%)
BMI	18.5 (SD:2.1)	25.5 (SD:3.9)	18.1 (SD:1.9)	25.3 (SD:4.1)	24.7 (SD:4.6)
Living status before admission					
Alone	49 (55.7%)	523 (46.7%)	187 (70.3%)	1636 (72.7%)	2395 (64.3%)
Together	39 (44.3%)	590 (52.7%)	78 (29.3%)	602 (26.8%)	1309 (35.2%)
Missing	0	6 (0.5%)	1 (0.4%)	11 (0.5%)	18 (0.5%)
Number of underlying diseases					
≤ 1	33 (37.5%)	396 (35.4%)	107 (40.2%)	862 (38.3%)	1398 (37.6%)
≥ 2	55 (62.5%)	723 (64.6%)	159 (59.8%)	1387 (61.7%)	2324 (62.4%)
CS					
≤ 2	62 (70.5%)	783 (70.0%)	187 (70.3%)	1736 (77.2%)	2768 (74.4%)
≥ 3	26 (29.5%)	336 (30.0%)	79 (29.7%)	511 (22.7%)	952 (25.6%)
Missing	0	0	0	2 (0.1%)	2 (0.1%)
DRS					
≤ 2	61 (69.3%)	842 (75.2%)	170 (63.9%)	1601 (71.2%)	2674 (71.8%)
≥ 3	27 (30.7%)	273 (24.4%)	93 (35.0%)	641 (28.5%)	1034 (27.8%)
Missing	0	4 (0.4%)	3 (1.1%)	7 (0.3%)	14 (0.4%)
RISE					
≤ 2	34 (38.6%)	292 (26.1%)	77 (28.9%)	503 (22.4%)	906 (24.3%)
≥ 3	53 (60.2%)	821 (73.4%)	188 (70.7%)	1736 (77.2%)	2798 (75.2%)
Missing	1 (1.1%)	6 (0.5%)	1 (0.4%)	10 (0.4%)	18 (0.5%)
ABS					
0	63 (71.6%)	818 (73.1%)	202 (75.9%)	1801 (80.1%)	2884 (77.5%)
≥ 1	25 (28.4%)	300 (26.8%)	64 (24.1%)	444 (19.7%)	833 (22.4%)
Missing	0	1 (0.1%)	0	4 (0.2%)	5 (0.1%)
CPS					
≤ 2	64 (72.7%)	772 (69.0%)	184 (69.2%)	1701 (75.6%)	2721 (73.1%)
≥ 3	24 (27.3%)	340 (30.4%)	80 (30.1%)	530 (23.6%)	974 (26.2%)
Missing	0	7 (0.6%)	2 (0.8%)	18 (0.8%)	27 (0.7%)
Number of behavioral-cognitive problems					
0	26 (29.5%)	425 (38.0%)	91 (34.2%)	997 (44.3%)	1539 (41.3%)
1	28 (31.8%)	259 (23.1%)	61 (22.9%)	526 (23.4%)	874 (23.5%)
2	8 (9.1%)	177 (15.8%)	51 (19.2%)	321 (14.3%)	557 (15.0%)
3	16 (18.2%)	135 (12.1%)	32 (12.0%)	215 (9.6%)	398 (10.7%)
4	6 (6.8%)	92 (8.2%)	21 (7.9%)	124 (5.5%)	243 (6.5%)
5	4 (4.5%)	31 (2.8%)	10 (3.8%)	64 (2.8%)	109 (2.9%)
Missing	0	0	0	2 (<0.1%)	2 (<0.1%)


Data is shown as mean (Standard deviation) or as number (percentage); Abbreviations: BMI (body mass index), CS (communication scale), DRS (depressive rating scale), RISE (revised index of social engagement), ABS (aggressive behavior scale), CPS (cognitive performance scale).

In total, 88 male residents (7.3%) and 266 (10.6%) female residents were malnourished at admission. In women, prevalence rates of malnutrition ranged from 12.5–13.5% for each of the behavior and cognitive problems. In male residents, malnutrition was lowest (6.5%) in residents with cognitive problems and highest (10.4%) in residents with low social engagement (table 2).

**Table 2:** Odds Ratio's for CPS, CS, DRS, RISE, ABS and total number of behavioral-cognitive problems in relation to malnutrition, stratified by gender

	**Male N=1297 (32.4%)**			**Female N=2515 (67.6%)**		
	**Malnutrition prevalence**	**Bivariate analyses**	Full model*	**Malnutrition prevalence**	**Bivariate analyses**	Full model*
CS						
≤ 2	7.3%	Ref.	Ref.	10.2%	Ref.	Ref.
≥ 3	7.2%	0.98 (0.61–1.57)	1.08 (0.57–2.05)	13.4%	1.44 (1.08–1.90)	1.21 (0.83–1.75)
DRS						
≤ 2	6.8%	Ref.	Ref.	9.6%	Ref.	Ref.
≥ 3	9.0%	1.37 (0.85–2.19)	1.35 (0.80–2.29)	12.7%	1.37 (1.04–1.79)	1.22 (0.90–1.66)
RISE						
≥ 3	6.1%	Ref.	Ref.	9.8%	Ref.	Ref.
≤ 2	10.4%	1.80 (1.15–2.83)	1.80 (1.13–2.86)	13.5%	1.41 (1.07–1.88)	1.33 (0.99–1.78)
ABS						
0	7.2%	Ref.	Ref.	10.1%	Ref.	Ref.
≥ 1	7.6%	1.08 (0.67–1.75)	0.96 (0.55–1.67)	12.5%	1.29 (0.95–1.73)	1.04 (0.73–1.48)
CPS						
≤ 2	7.7%	Ref. †	Ref.	9.7%	Ref. †	Ref.
≥ 3	6.5%	0.85 (0.52–1.39)	0.77 (0.40–1.48)	13.5%	1.40 (1.06–1.85)	1.06 (0.73–1.54)
Number of behavioral-cognitive problems						
0	26 (5.8%)	Ref. †'	Ref.	91 (8.4%)	Ref. †	Ref.
1	28 (9.8%)	1.76 (1.01–3.07)	1.79 (1.02–3.12)	61 (10.4%)	1.27 (0.90–1.78)	1.24 (0.88–1.75)
2	8 (4.3%)	0.74 (0.33–1.66)	0.77 (0.34–1.73)	51 (13.7%)	1.74 (1.21–2.51)	1.72 (1.19–2.49)
3	16 (10.6%)	1.96 (1.02–3.76)	2.11 (1.09–4.08)	32 (13.0%)	1.60 (1.03–2.46)	1.58 (1.02–2.45)
4	6 (6.1%)	1.06 (0.43–2.65)	1.13 (0.44–2.83)	21 (14.5%)	1.88 (1.13–3.13)	1.82 (1.08–3.05)
5	4 (11.4%)	2.24 (0.73–6.86)	2.37 (0.77–7.30)	10 (13.5%)	1.71 (0.85–3.43)	1.68 (0.83–3.41)
P-value test for trend		0.291	0.188		0.001	0.001


Data is shown as percentage or odds ratios with 95% confidence interval; Abbreviations: CS (communication scale), DRS (depressive rating scale), RISE (revised index of social engagement), ABS (aggressive behavior scale), CPS (cognitive performance scale) N.A. (Not applicable); * For behavioral cognitive scales, model consist of: CS, DRS, RISE, ABS, CPS, age category (≤ 89 years vs. ≥ 90 years), number of comorbidities (≤1 vs. ≥2) and living status before admission (alone vs. together). For total number of behavioral-cognitive problems, model consist of total number of behavioral-cognitive problems, age category (≤ 89 years vs. ≥ 90 years), number of comorbidities (≤1 vs. ≥2) and living status before admission (alone vs. together); † significant interaction term (p<0.10) between behavioral cognitive problem and gender

Bivariate analyses showed that CS, DRS, RISE and CPS were significantly associated with an increased odds of being malnourished in female residents. Adjustments for age, number of comorbidities and living status before admission had only minor influence (see appendix 2). In the full model associations attenuated, but a trend for higher prevalence of malnutrition in residents with behavior and cognitive problems remained. In men, only RISE was significantly associated with malnutrition. In women, a clear trend (p-value <0.001) was seen between having multiple behavioral-cognitive problems and prevalence of malnutrition. In male residents, this trend was not seen but power was low in this group.

### Prospective analysis

As depicted in table 3, data of 4978 residents were available for the prospective analysis, 1826 with an ‘admission assessment’ (‘newly-admitted’ residents) and 3152 with a ‘delayed first assessment’ as reference assessment (‘existing’ residents). Most residents were women (71.3%), aged <90 years (78.5%), had ≥2 diseases (61.6%), and a mean BMI of 25.7 kg/m2. Nearly 1 out of 3 residents had depressive symptoms based on DRS (31.0%), other behavioral-cognitive problems ranged between 25.5–28.0%.

**Table 3:** Characteristics included participants for prospective analysis stratified by gender, assessment type and their nutritional status

	**‘Admission assessments’ in ‘newly-admitted’ residents' N=1826**				**‘Delayed first assessments’ in ‘existing’ residents N=3152**				
	**Male N=599 (41.9)**		**Female N=1227 (28.8)**		**Male N=831 (58.1)**		**Female N=2321 (71.2)**		**Total N=4978**
	**Became Malnourished N=41 (6.8)**	**Stayed well-nourished N=558 (93.2)**	**Became Malnourished N=103 (8.3)**	**Stayed well-nourished N=1124 (91.7)**	**Became Malnourished N=65 (7.8)**	**Stayed well-nourished N=766 (92.2)**	**Became Malnourished N=234 (10.1)**	**Stayed well-nourished N=2087 (89.9)**	
Age (years)	84.3 (6.5)	81.7 (6.6)	83.5 (6.5)	83.7 (6.8)	82.2 (7.0)	82.5 (7.5)	86.5 (6.7)	84.8 (6.8)	83.9 (7.0)
≤ 89 years	31 (75.6)	500 (89.6)	83 (80.6)	909 (80.9)	56 (86.2)	636 (83.0)	149 (63.7)	1542 (73.9)	3906 (78.5)
≥ 90 years	10 (24.4)	58 (10.4)	20 (19.4)	215 (19.1)	9 (13.8)	130 (17.0)	85 (36.3)	545 (26.1)	1072 (21.5)
BMI (kg/m^2^)	23.0 (2.4)	25.8 (3.9)	21.8 (2.1)	25.7 (4.5)	22.8 (2.6)	25.8 (3.8)	22.3 (2.5)	26.3 (4.6)	25.7 (4.4)
Living status before admission									
Alone	18 (43.9)	278 (49.8)	74 (71.8)	802 (71.4)	36 (55.4)	375 (49.0)	170 (72.6)	1451 (69.5)	3204 (64.4)
Together	23 (56.1)	278 (49.8)	28 (27.2)	316 (28.1)	28 (43.1)	385 (50.3)	62 (26.5)	605 (29.0)	1725 (34.7)
Missing	0	2 (0.4)	1 (1.0)	6 (0.5)	1 (1.5)	6 (0.8)	2 (0.9)	31 (1.5)	49 (1.0)
Diseases									
≤ 1	16 (39.0)	208 (37.7)	36 (35.0)	436 (38.8)	25 (38.5)	292 (38.1)	93 (39.7)	805 (38.6)	1911 (38.4)
≥ 2	25 (61.0)	350 (62.7)	67 (65.0)	688 (61.2)	40 (61.5)	474 (61.9)	141 (60.3)	1282 (61.4)	3067 (61.6)
CS									
≤ 2	31 (75.6)	403 (72.2)	62 (60.2)	873 (77.7)	34 (52.3)	537 (70.1)	158 (67.5)	1544 (74.0)	3642(73.2)
≥ 3	10 (24.4)	155 (27.8)	41 (39.8)	250 (22.2)	31 (47.7)	227 (29.6)	76 (32.5)	543 (26.0)	1333 (26.8)
Missing	0	0	0	1 (0.1)	0	2 (0.3)	0	0	3 (0.1)
DRS									
≤ 2	32 (78.0)	427 (76.5)	72 (69.9)	804 (71.5)	34 (52.3)	539 (70.4)	154 (65.8)	1357 (65.0)	3419 (68.7)
≥ 3	9 (22.0)	127 (22.8)	30 (29.1)	318 (28.3)	31 (47.7)	225 (29.4)	80 (34.2)	725 (34.7)	1545 (31.0)
Missing	0	4 (0.7)	1 (1.0)	2 (0.2)	0	2 (0.3)	0	5 (0.2)	14 (0.3)
RISE									
≤ 2	12 (29.3)	132 (23.7)	24 (23.3)	241 (21.4)	30 (46.2)	230 (30.0)	71 (30.3)	527 (25.3)	1267 (25.5)
≥ 3	29 (70.7)	422 (75.6)	79 (76.7)	879 (78.2)	35 (53.8)	534 (69.7)	163 (69.7)	1554 (74.5)	3695 (74.2)
Missing	0	4 (0.7)	0	4 (0.4)	0	2 (0.3)	0	6 (0.3)	16 (0.3)
ABS									
0	32 (78.0)	400 (71.7)	73 (70.9)	884 (78.6)	30 (46.2)	504 (65.8)	169 (72.2)	1566 (75.0)	3658 (73.5)
≥ 1	9 (22.0)	158 (28.3)	30 (29.1)	238 (21.2)	35 (53.8)	260 (33.9)	65 (27.8)	521 (25.0)	1316 (26.4)
Missing	0	0	0	2 (0.2)	0	2 (0.3)	0	0	4 (0.1)
CPS									
≤ 2	26 (63.4)	388 (69.5)	68 (66.0)	848 (75.4)	36 (55.4)	537 (70.1)	158 (67.5)	1498 (71.8)	3559 (71.5)
≥ 3	15 (36.6)	166 (29.7)	34 (33.0)	268 (23.8)	29 (44.6)	225 (29.4)	76 (32.5)	580 (27.8)	1393 (28.0)
Missing	0	4 (0.7)	1 (1.0)	8 (0.7)	0	4 (0.5)	0	9 (0.4)	26 (0.5)
Number of behavioral-cognitive problems									
0	15 (36.6)	224 (40.1)	36 (35.0)	494 (44.0)	11 (16.9)	257 (33.6)	77 (32.9)	790 (37.9)	1904 (38.2)
1	8 (19.5)	127 (22.8)	25 (24.3)	259 (23.0)	11 (16.9)	179 (23.4)	59 (25.2)	481 (23.0)	1149 (23.1)
2	10 (24.4)	80 (14.3)	13 (12.6)	168 (14.9)	11 (16.9)	129 (16.8)	37 (15.8)	332 (15.9)	780 (15.7)
3	5 (12.2)	68 (12.2)	13 (12.6)	117 (10.4)	11 (16.9)	102 (13.3)	22 (9.4)	257 (12.3)	595 (12.0)
4	3 (7.3)	48 (8.6)	11 (10.7)	56 (5.0)	15 (23.1)	61 (8.0)	26 (11.1)	155 (7.4)	375 (7.5)
5	0	11 (2.0)	5 (4.9)	29 (2.6)	6 (9.2)	36 (4.7)	13 (5.6)	72 (3.4)	172 (3.5)
missing	0	0	0	1 (0.1)	0	2 (0.3)	0	0	3 (0.1)


All characteristics are number with percentage except age (years) and BMI (kg/m2) which are presented as mean with standard deviation. Abbreviations: BMI (body mass index), CS (communication scale) DRS (depressive rating scale), RISE (revised index of social engagement), ABS (aggressive behavior scale), CPS (cognitive performance scale).

Total follow-up of all participants was 7104 residents' years with a median individual follow up of 357 days. During this period, 17217 follow-up measurements were performed (including last assessment). Incident malnutrition occurred in 106 (7.4%) male residents and 337 (9.5%) female residents. Incident malnutrition per follow-up year was lowest in ‘existing’ male residents (0.052 per follow-up year) and highest in ‘newly-admitted’ female residents (0.071 per follow-up year).

As shown in table 4, none of the behavior and cognitive problems was significantly associated with incident malnutrition in ‘newly-admitted’ male residents (n=599). In contrast, in ‘existing’ male residents (n=831), all behavioral-cognitive problems were associated with incident malnutrition in the bivariate analyses. Like in the cross-sectional analysis, adjustments for age, number of comorbidities and living status before admission had little influence (appendix 2). In the full model a clear trend was seen for an increased risk of becoming malnourished for all behavior and cognitive problems, although only for DRS this was statistically significant (HR:1.72 [95%CI: 1.00–2.96]).

**Table 4:** Hazard ratio's for CPS, CS, DRS, RISE, ABS and total number of behavioral-cognitive problems in relation to malnutrition, stratified by gender and assessment type

		**‘Admission assessments’ in ‘newly-admitted’ residents**		**‘Delayed first assessments’ in ‘existing’ residents**	
		**Bivariate analyses**	Full model*	**Bivariate analyses**	Full model*
CS	ref ≤ 2				
Male	≥ 3	1.02 (0.50–2.10)†,*	0.69 (0.28–1.70)	2.94 (1.79–4.84)*	1.67 (0.86–3.26)
Female	≥ 3	2.46 (1.65–3.67)†	2.32 (1.40–3.86)	1.83 (1.39–2.41)	1.44 (0.97–2.14)
DRS	ref ≤ 2				
Male	≥ 3	1.14 (0.54–2.41)*	1.05 (0.48–2.30)	2.55 (1.56–4.18)†,*	1.72 (1.00–2.96)
Female	≥ 3	1.15 (0.75–1.76)	0.76 (0.46–1.24)	1.11 (0.85–1.46)t	0.94 (0.70–1.26)
RISE	ref ≥ 3				
Male	≤ 2	1.27 (0.65–2.50)	1.39 (0.70–2.76)	1.92 (1.18–3.12)	1.40 (0.84–2.33)
Female	≥ 2	1.26 (0.80–2.00)	1.02 (0.62–1.67)	1.49 (1.12–1.96)	1.27 (0.95–1.70)
ABS	ref = 0				
Male	≥ 1	0.80 (0.38–1.70)*,†	0.67 (0.30–1.51)	2.60 (1.59–4.24)*,†	1.54 (0.86–2.74)
Female	≥ 1	1.61 (1.05–2.47)†	1.29 (0.80–2.08)	1.39 (1.04–1.85)t	1.14 (0.82–1.58)
CPS	ref ≤ 2				
Male	≥ 3	1.47 (0.77–2.79)	2.17 (0.95–4.97)	2.75 (1.67–4.52)	1.42 (0.72–2.80)
Female	≥ 3	1.78 (1.17–2.68)	1.06 (0.63–1.78)	1.74 (1.32–2.29)	1.33 (0.90–1.98)
Number of behavioral-cognitive problems					
*Male*					
0		Ref.†,*	Ref.	Ref.†,*	Ref.
1		0.81 (0.34–1.92)	0.81 (0.34–1.93)	1.76 (0.76–4.07)	1.78 (0.77–4.12)
2		1.64 (0.73–3.66)	1.67 (0.74–3.80)	2.36 (1.02–5.45)	2.48 (1.06–5.77)
3		1.71 (0.61–4.82)	1.70 (0.60–4.84)	3.42 (1.47–7.93)	3.42 (1.46–8.01)
4		1.08 (0.31–3.76)	1.05 (0.30–3.71)	8.80 (3.99–19.45)	9.88 (4.40–22.21)
5		N.A.	N.A.	5.94 (2.18–16.21)	6.03 (2.18–16.72)
P-value test for trend		0.564	0.548	<0.001	<0.001
Number of behavioral-cognitive problems					
*Female*					
0		Ref.t	Ref.	Ref.t	Ref.
1		1.36 (0.82–2.28)	1.33 (0.80–2.23)	1.46 (1.04–2.05)	1.42 (1.01–1.99)
2		1.11 (0.59–2.10)	1.05 (0.55–2.00)	1.42 (0.96–2.10)	1.49 (1.00–2.22)
3		1.71(0.90–3.23)	1.66 (0.87–3.17)	1.25 (0.77–2.03)	1.29 (0.79–2.10)
4		3.54 (1.79–7.00)	3.55 (1.79–7.05)	2.58 (1.65–4.03)	2.71 (1.70–4.32)
5		2.97 (1.16–7.58)	2.78 (1.07–7.24)	2.79(1.55–5.04)	3.06 (1.67–5.61)
P-value test for trend		0.001	0.001	<0.001	<0.001


Data is shown as Hazard ratios with 95% confidence interval; Abbreviations: CS (communication scale) DRS (depressive rating scale), RISE (revised index of social engagement), ABS (aggressive behavior scale), CPS (cognitive performance scale), N.A. (Not applicable); * For behavioral cognitive scales, model consist of: CS, DRS, RISE, ABS, CPS, age category (≤ 89 years vs. ≥ 90 years), number of comorbidities (≤1 vs. ≥2) and living status before admission (alone vs. together). For total number of behavioral-cognitive problems, model consist of total number of behavioral-cognitive problems, age category (≤ 89 years vs. ≥ 90 years), number of comorbidities (≤1 vs. ≥2) and living status before admission (alone vs. together); † significant interaction term (p<0.10) between behavioral cognitive problem and gender * significant interaction term (p<0.10) between behavioral cognitive problem and type of first assessment

In women, small differences were seen between ‘newly-admitted’ (n=1227) and ‘existing’ residents (n=2321) (Table 4). In the bivariate analysis, all behavioral-cognitive problems (except DRS in both groups and RISE in ‘newly-admitted’ female residents) were significantly associated with incident malnutrition. In the full model, only communication problems remained independently associated with becoming malnourished in ‘newly-admitted’ female residents (CS HR:2.32 [95%CI: 1.40–3.86]). No clear trend was seen for the other behavior and cognitive problems.

Having more behavior and cognitive problems was related to a higher risk of becoming malnourished. In ‘newly-admitted’ male residents this was not observed, but in ‘existing’ male residents as well in both female groups this was observed (p-value test for trend <0.001). Especially in ‘existing’ male residents high HRs were observed, with HR's ranging from 6.03–9.88 for 4–5 behavior and cognitive problems. In female residents, we found an increased risk of malnutrition in residents with 1–3 behavioral-cognitive problems (HR's ranging from 1.05–1.66), and this was even stronger for residents with 4–5 problems (HR's ranging from 2.71–3.55) (Table 4).

## Discussion

Our results show that approximately 9.5% of all LTCF residents with behavior and cognitive problems are malnourished at admission and another 8.8% become malnourished during stay. The more behavior and cognitive problems, the higher the risk of developing malnutrition.

Our data indicate that behavior and cognitive problems are related with incident malnutrition in both men and women. For male residents in the cross-sectional and in the prospective analyses of ‘newly-admitted’ residents, no clear relation with malnutrition was seen for most behavior-cognitive problems. However, among ‘existing’ residents, a clear relation between behavior-cognitive problems and malnutrition was observed, indicating that the manifestation is later in men. In female residents, such a difference between ‘newly-admitted’ and ‘existing’ residents was not seen and their risk was more stable over time.

As was seen in the cross-sectional analyses, male residents are entering LTCFs in a better nutritional state compared to women. We hypothesize that this could be explained by the home situation before admission; women traditionally prepare meals, and take care of their husbands with behavioral-cognitive problems. The other way around is usually more problematic, as older men generally lack cooking skills (36). Male residents were more frequently living with a partner or children and might therefore have been better taken care of before admission to LTCF, and this may explain the lower malnutrition rates at admission. This effect seemed to decrease over time and malnutrition developed in male ‘existing’ residents as well. We thus suggest to take preventative measures in the first year after admission to LTCF.

Our statistical models showed attenuating associations when all behavior and cognitive problems were included. The attenuation of all effect-sizes within a model can only be explained by the presence of multiple behavioral-cognitive problems within one person. Indeed, most residents with behavior and cognitive problems had more than one problem, which reflects reality; the more problems, the higher the risk to be become malnourished. This may explain the observed loss of significance after including all problems in the model. Interventions to prevent malnutrition should therefore especially focus on residents with multiple behavior and cognitive problems.

The prevalence of malnutrition at admission in our sample was 9.5% which was lower compared to a recent meta-analysis (34) where 17.5% was malnourished. However, in the meta-analysis most studies assessed malnutrition during stay, which also includes long-stay residents. In addition, different definitions of malnutrition were used: the review was based on screening tool outcomes, while we used the ESPEN 2015 definition, which strongly relies on BMI. As BMI is relatively high in older adults, the ESPEN 2015 definition is thought to underestimate the prevalence of malnutrition (37). For incidence rates, the underestimation might be even stronger. During stay, residents with overweight/obesity are not likely to lose weight to the extent that they fall below the cut-off points from the ESPEN 2015 definition. Therefore, we suggest to implement the recently published GLIM criteria (38) in the InterRAI minimum dataset form in the future as these criteria rely less on BMI (37).

We decided not to adjust our analyses for BMI at first measurement. Within the ESPEN definition, a high BMI is protective for developing malnutrition in the future as one can only be categorized as malnourished by having a low age-specific BMI combined with recent weight loss, or by having a very low BMI (<18.5 kg/m^2^). In accordance with this definition, weight loss alone will not trigger these cut-off values in older adults with a high BMI. By adjusting for BMI at baseline, a potential real protective effect of BMI would have been removed, and lead to over-adjustment.

To the best of our knowledge, this is the first study in a residential-care setting on the prospective associations between behavioral-cognitive problems and malnutrition. Two previous prospective studies in the community and sheltered-house setting reported significant associations between poor cognition and malnutrition (39, 40). In contrast, one study showed no such association but in this study a relatively high cut-off value for cognitive problems was used (MMSE<24) (41). Previous research on the association between depression and malnutrition showed contrasting results as a significant association was seen in Schilp et al. (41), but not in Mamhidir et al. (40). Neither study adjusted for other behavioral-cognitive problems, while we have shown that the combination of problems increases the risk.

For most behavior and cognitive problems, a stronger relation with malnutrition was seen in the prospective analysis compared to the cross-sectional analysis. This probably is because behavioral-cognitive problems are progressive over time (42, 43, 44), and this is thought to directly affect the risk of malnutrition i.e. the worse the condition of the resident, the higher the malnutrition risk. At admission, most residents still have mild or moderate cognitive problems (45) and consequences regarding malnutrition can easily be addressed by regular care (46). However, during stay, residents are known to further lose cognitive functions and to become more care dependent (47). Severe cognitive decline or dementia can even result in swallowing and chewing problems, and refusal to eat and drink (48). These problems require extensive individualized care. For daily care, we suggest to routinely check whether standard nutritional care is still sufficient in the light of someone's cognitive decline or if more individualized care is needed.

### Strengths and weaknesses

Strong points of our study were the large number of residents and large total follow-up years. The mean follow-up period per person of approximately one year may be relatively short for a prospective study on malnutrition, but is in accordance with the average length of stay in a LTCF. We excluded residents with end-stage disease, i.e. terminally ill residents with a life expectancy <6 months, at their first or last measurement to exclude residents with incurable malnutrition. In general, treatment of malnutrition in nursing homes, either curative or palliative, should be guided by the stage of life of residents and their personal wishes to be treated (49).

A weakness of our data may be the relative long interval between measurements. Unobserved changes in nutritional status may have taken place between two measurements. On the other hand, nutritional status of most persons is relatively stable. Furthermore, we used dichotomized outcomes for all behavior-cognitive problems as this better reflects the clinical situation. Note that for ABS four categories are described in the validation paper (0, 1–2, 3–5, ≥6) but we did not have enough power in the 3–5 and ≥6 category, hence we decided to dichotomize it in ABS problems present no/yes (ABS=0 vs. ABS ≥1) (29). Lastly, we used cognitive status at first available measurement as independent variable. Residents without behavior and cognitive problems at first assessment could have developed these problems during their stay. This could have led to an underestimation of the effect sizes.

## Conclusion

Residents in LTCF with behavior and cognitive problems are at an increased risk of being malnourished at admission and to become malnourished during stay, and more specifically during long-term stay. Male residents with behavior and cognitive problems are in better nutritional condition at admission and seem to develop malnutrition in a later stage compared to female residents. This emphasizes the need of early identification and treatment of malnutrition in residents with behavioral-cognitive problems.

## Availability of data and materials

The datasets generated and/or analysed during the current study are not publicly available due to restrictions of the data owner (InterRAI) but are available from the corresponding author on reasonable request.

## Electronic Supplementary Material

Communication scale

Appendix 1, Odds/Hazard ratio's for CPS, CS, DRS, RISE, ABS and total number of behavioral-cognitive problems in relation to malnutrition, stratified by gender
