# Supplementary figures and images for: Behavioral and Cognitive Problems as Determinants of Malnutrition in Long-Term Care Facilities, a Cross-Sectional and Prospective Study
Source: J Nutr Health Aging. 2022 Jul 26;26(8):749–59. doi: 10.1007/s12603-022-1827-3 (PMC12280647; doi:10.1007/s12603-022-1827-3)

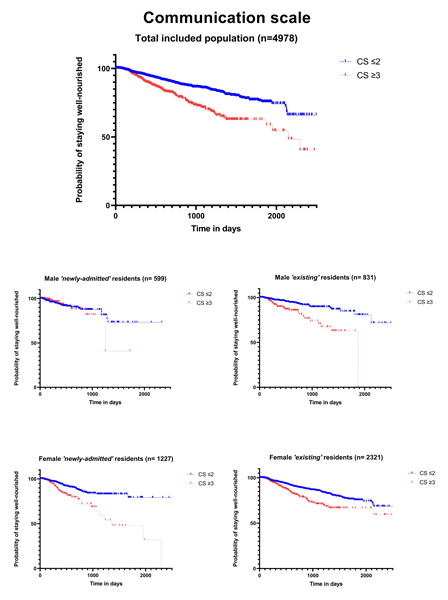

Supplement: Supplementary file 1 — Communication scale [file mmc1.jpg]
