# Supplementary material for: Behavioral and Cognitive Problems as Determinants of Malnutrition in Long-Term Care Facilities, a Cross-Sectional and Prospective Study
Source: J Nutr Health Aging. 2022 Jul 26;26(8):749–59. doi: 10.1007/s12603-022-1827-3 (PMC12280647; doi:10.1007/s12603-022-1827-3)
Supplement: Supplementary file 2 — Appendix 1, Odds/Hazard ratio's for CPS, CS, DRS, RISE, ABS and total number of behavioral-cognitive problems in relation to malnutrition, stratified by gender [file mmc2.docx]

Appendix 1, Odds/Hazard ratio’s for CPS, CS, DRS, RISE, ABS and total number of behavioral-cognitive problems in relation to malnutrition, stratified by gender

|  | Cross-sectional  analysis | | Prospective analysis | | | |
| --- | --- | --- | --- | --- | --- | --- |
|  | Admission assessment | | ‘Admission assessments’ in *‘newly-admitted’* residents | | ‘Delayed first assessments’ in *‘existing’* residents | |
|  | Men  N=1297 (32.4%) | Women N=2515 (67.6%) | Men  N=599 (41.9%) | Women N=1227 (28.8%) | Men  N=831 (58.1%) | Women N=2321 (71.2%) |
|  | Adjusted Odds ratio’s* | Adjusted  Odds ratio’s* | Adjusted hazard ratio* | Adjusted hazard ratio* | Adjusted hazard ratio* | Adjusted hazard ratio* |
| **CS**  ≤ 2 vs. ≥ 3 | 1.03 (0.64-1.67) | 1.41 (1.06-1.87) | 1.01 (0.49-2.08) | 2.46 (1.66-3.63) | 2.98 (1.80-4.95) | 1.87  (1.40-2.49) |
| **DRS**  ≤ 2 vs. ≥ 3 | 1.42 (0.88-2.30) | 1.35 (1.02-1.77) | 1.03  (0.48-2.20) | 1.10  (0.71-1.70) | 2.59 (1.57-4.28) | 1.13 (0.86-1.49) |
| **RISE**  ≥ 3 vs. ≤ 2 | 1.79  (1.14-2.81) | 1.40  (1.06-1.87) | 1.34 (0.68-2.64) | 1.24 (0.78-1.96) | 1.91 (1.17-2.78) | 1.41 (1.07-1.87) |
| **ABS**  0 vs. ≥ 1 | 1.13 (0.70-1.83) | 1.27 (0.93-1.72) | 0.84 (0.39-1.78) | 1.57 (1.02-2.42) | 2.65 (1.61-4.35) | 1.44 (1.08-1.93) |
| **CPS**  ≤ 2 vs. ≥ 3 | 0.91 (0.56-1.49) | 1.35 (1.01-1.80) | 1.49 (0.78-2.86) | 1.74 (1.14-2.65) | 2.82 (1.70-4.67) | 1.80 (1.34-2.41) |
| **Number of behavioral-cognitive problems**  **0**  **1**  **2**  **3**  **4**  **5** | Ref.  1.78 (1.02-3.12) 0.77 (0.34-1.73) 2.11 (1.09-4.08) 1.13 (0.45-2.83) 2.37 (0.77-7.30) | Ref.  1.24 (0.88-1.75) 1.72 (1.19-2.49) 1.58 (1.02-2.45) 1.82 (1.08-3.05) 1.68 (0.83-3.41) | Ref.  0.81 (0.34-1.93) 1.67 (0.74-3.80) 1.70 (0.60-4.84) 1.05  (0.30-3.71)  N.A. | Ref.  1.33 (0.80-2.23)  1.05  (0.55-2.00)  1.66 (0.87-3.17)  3.55 (1.79-7.05)  2.78 (1.07-7.24) | Ref.  1.78 (0.77-4.12)  2.48 (1.06-5.77)  3.42 (1.46-8.01)  9.88 (4.40-22.21)  6.03 (2.18-16.72) | Ref.  1.42 (1.01-1.99)  1.49 (1.00-2.22)  1.29 (0.79-2.10)  2.71  (1.70-4.32)  3.06 (1.67-5.61) |

**Data is shown as percentage or odds/hazard ratios with 95% confidence interval.
Abbreviations: CS (communication scale), DRS (depressive rating scale), RISE (revised index of social engagement), ABS (aggressive behavior scale), CPS (cognitive performance scale) N.A. (Not applicable).**

*** adjusted for age category (≤** 89 **years vs. ≥ 90 years), number of comorbidities (≤1 vs. ≥2) and living status before admission (alone vs. together).**
